# Supplementary material for: Identifying the lungs as a susceptible site for allele-specific regulatory changes associated with type 1 diabetes risk
Source: Commun Biol. 2021 Sep 14;4:1072. doi: 10.1038/s42003-021-02594-0 (PMC8440780; doi:10.1038/s42003-021-02594-0)
Supplement: Supplementary file 2 — Supplementary Information [file 42003_2021_2594_MOESM2_ESM.pdf]

# Identifying the lungs as a susceptible site for allele-specific regulatory changes associated with type 1 diabetes risk

Daniel Ho[1*], Denis M. Nyaga[1*], William Schierding[1,2], Richard Saffery[3], Jo K. Perry[1,2], John A. Taylor[2,4], Mark H. Vickers[1], Andreas W. Kempa-Liehr[5], Justin M. O'Sullivan[1,2,4,6ǂ]

* These authors contributed equally

ǂ To whom correspondence should be addressed: justin.osullivan@auckland.ac.nz

1 Liggins Institute, The University of Auckland, Auckland, New Zealand

2 The Maurice Wilkins Centre, The University of Auckland, Auckland, New Zealand

3 Murdoch Children Research Institute, The University of Melbourne, Melbourne, Australia

4 School of Biological Sciences, The University of Auckland, Auckland, New Zealand

5 Department of Engineering Science, The University of Auckland, Auckland, New Zealand

6 MRC Lifecourse Epidemiology Unit, University of Southampton, United Kingdom

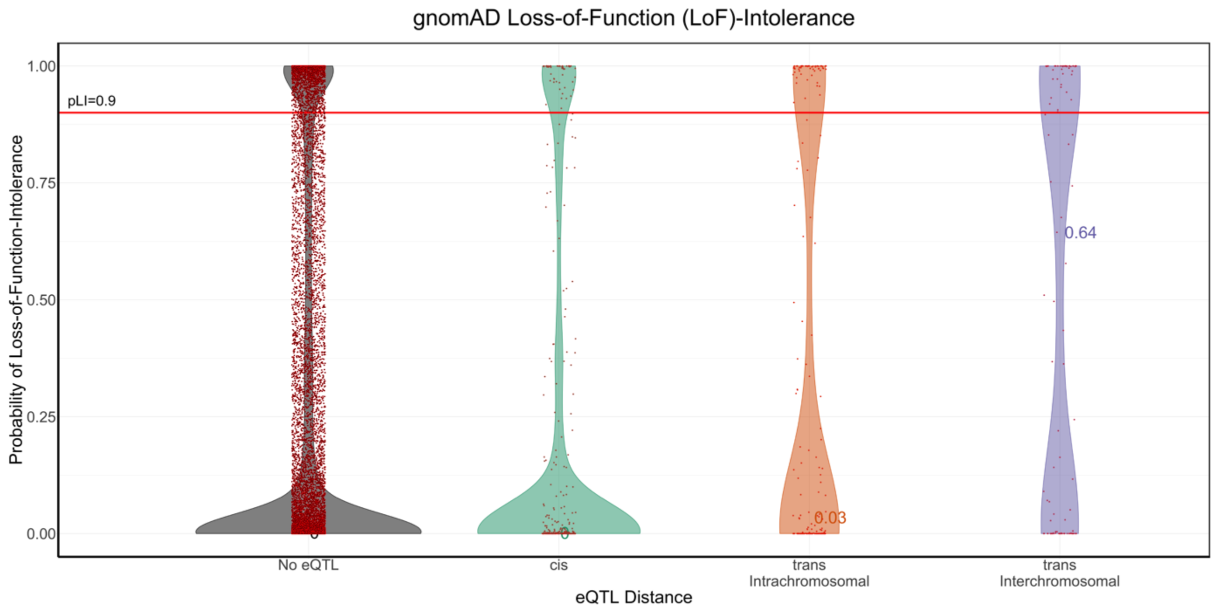


**Supplementary Figure 1. Loss of function analysis for spatially regulated genes.**
Analyses of the loss of function intolerance of the genes that are spatially regulated by T1D SNPs using the Genome Aggregation Database (gnomAD)[1]. *Trans*-regulated genes are less tolerant of inactivating mutations. No eQTLs – gnomAD genes with no significant eQTL associations with T1D SNPs; *cis* – genes with significant eQTL associations with T1D SNPs < 1 Mb window; *trans*-intrachromosomal – genes with significant eQTL associations with T1D SNPs > 1Mb but within the same chromosomes; *trans*-interchromosomal – genes with significant eQTL associations with T1D SNPs > 1Mb but on different chromosomes.

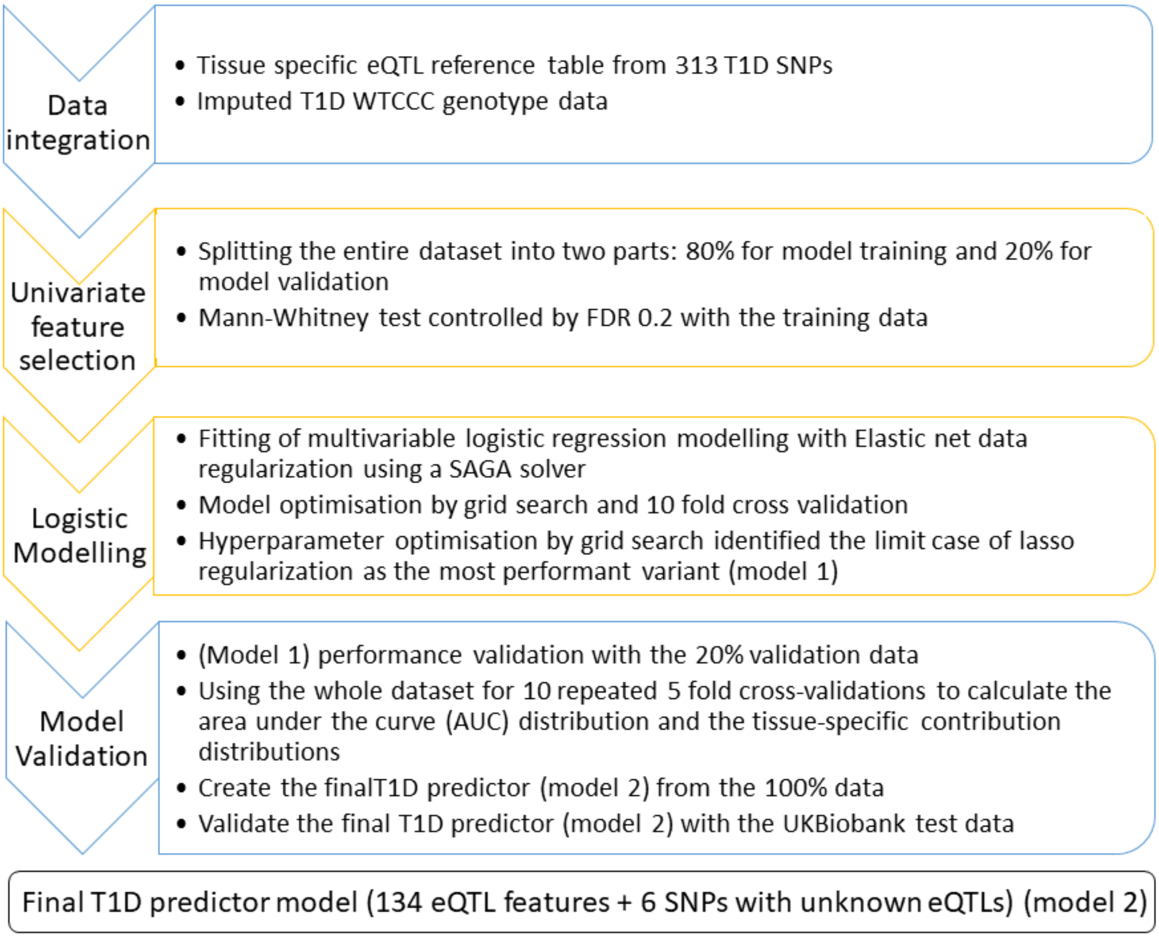


**Supplementary Figure 2.** Workflow for creating the T1D disease status predictor model using a regularised logistic regression model.

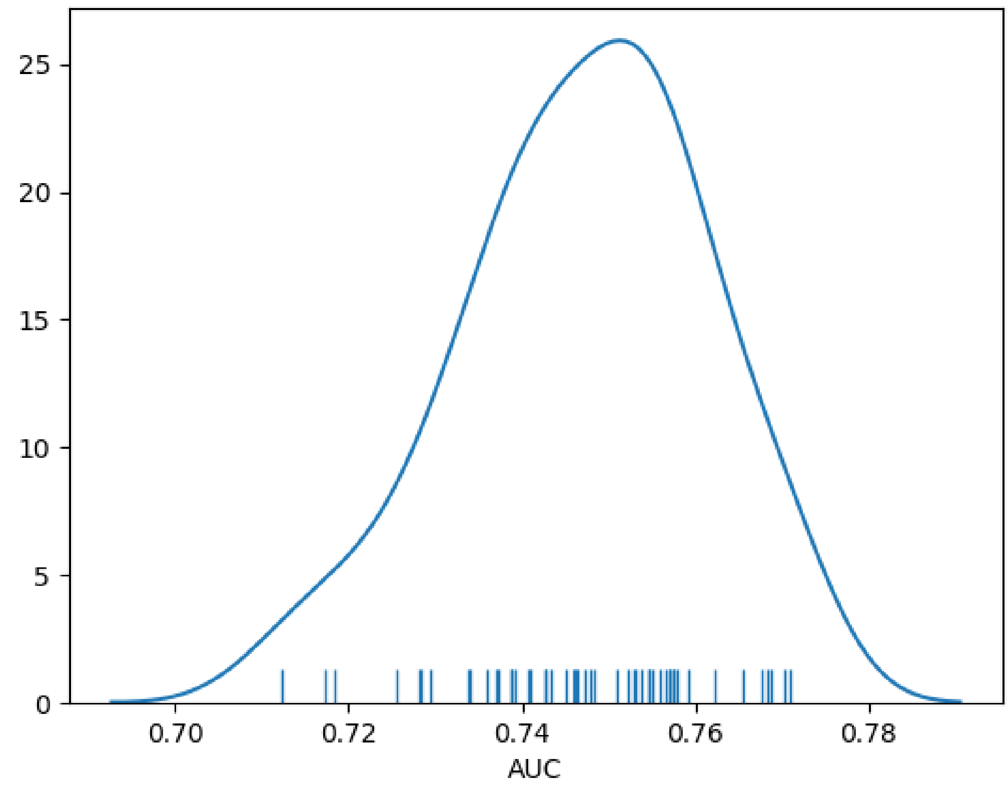


**Supplementary Figure 3. Kernel Density Estimate plot of AUC distribution created from the 10 repeated 5-fold cross-validations (50 T1D regularised prediction models with model 1's optimised hyperparameters).**

The AUC mean is 0.747 with the standard deviation of 0.14. [X axis: AUC, Y axis: frequency of AUC (%)]

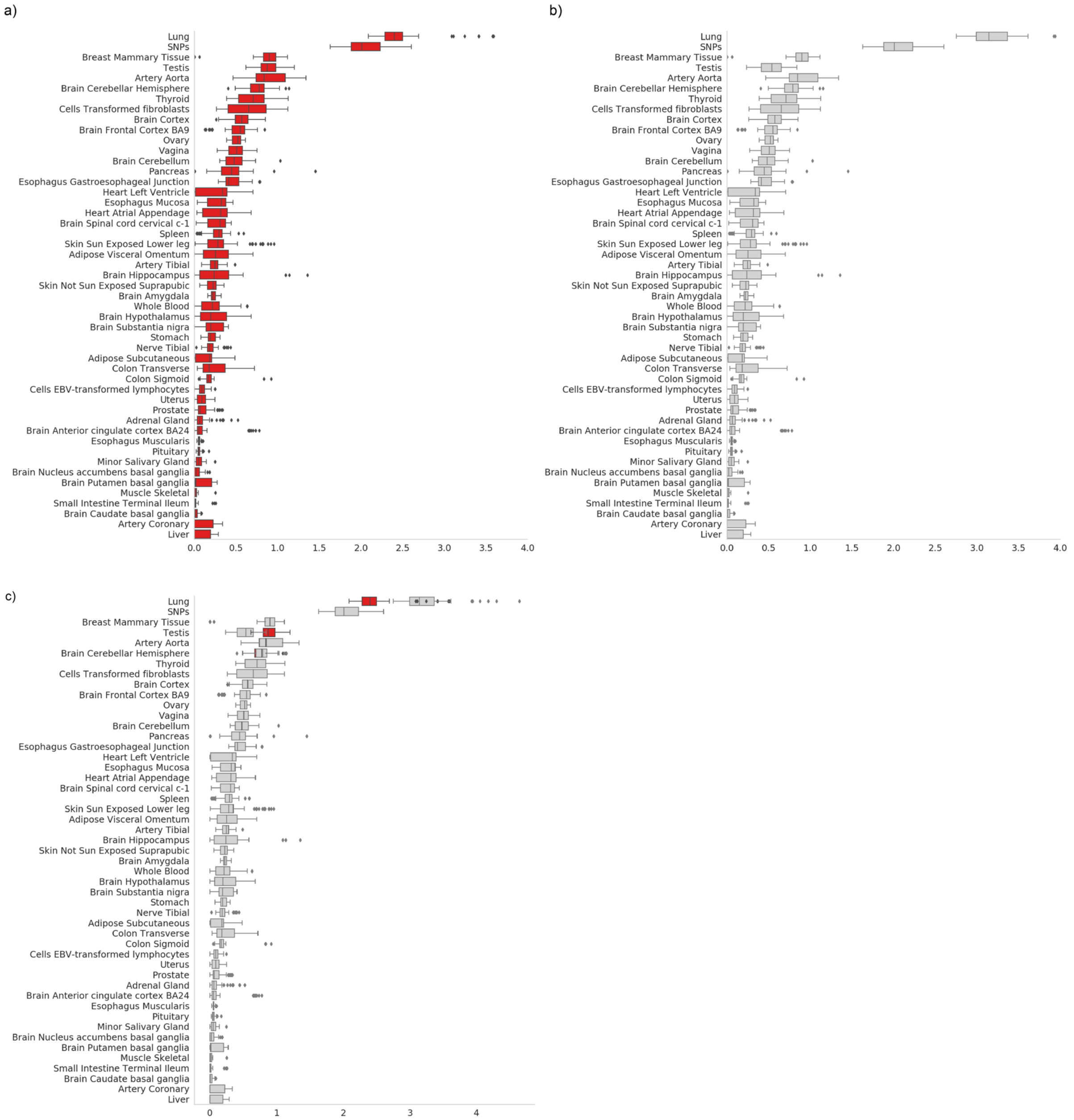


**Supplementary Figure 4. Tissue contributions of the T1D logistic lasso regression models.**

The tissue contributions with eQTL rs3087243 either at testis **(a)** or at lung **(b)** created from 10 repeated 5-fold cross-validations (50 predictors). **(c)** Overlap of **(a)** and **(b)** highlighting the lung and testes differences. The SNPs category denotes T1D-associated SNPs that are not eQTLs. The X axes are the total value of the model weights (no units).

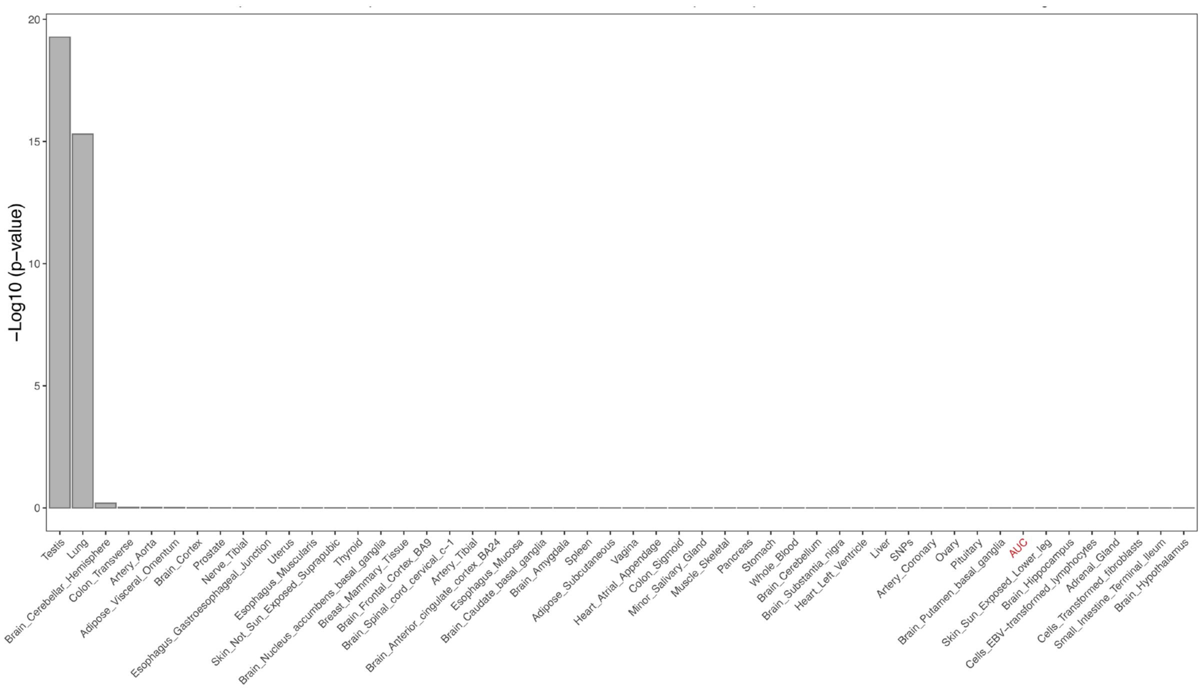


**Supplementary Figure 5. *P*-values (-log10) of the tissue-specific contribution and AUC differences of n=50 T1D predictor pairs with eQTL rs3087243 at testis or at lung evaluated by two-sided t-test.**

Most of the GTEx tissue-specific contributions and AUCs of the predictor pairs are not significantly different. However, the contributions at lung and testis have significant differences (p-value <0.01).

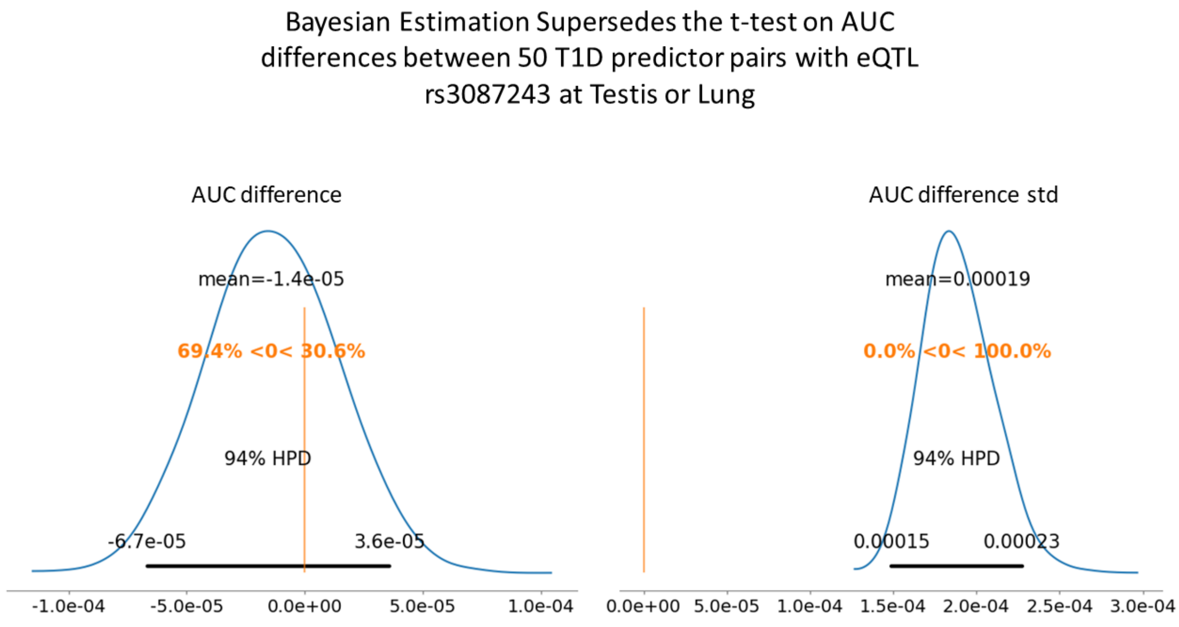


**Supplementary Figure 6. AUC difference distribution between 50 T1D regularised logistic regression predictor pairs with the eQTL rs3087243 either at testis or at lung evaluated by Bayesian estimation supersedes the t-test (1000 iterations of model simulation).**

The simulated AUC difference data mean is 1.4e-05 with the standard deviation of 0.00019. [Y axis: posterior distribution; X axis: Mean of AUC difference and Standard deviation of AUC differences; AUC; Area under the Curve. HPD; highest posterior density interval]. Informed prior was modelled as Student-T with mean ~ N(sample mean, sample std * 2), stand deviation ~ U(0,1) and degree of freedom ~ (Exp(1/29) +1).

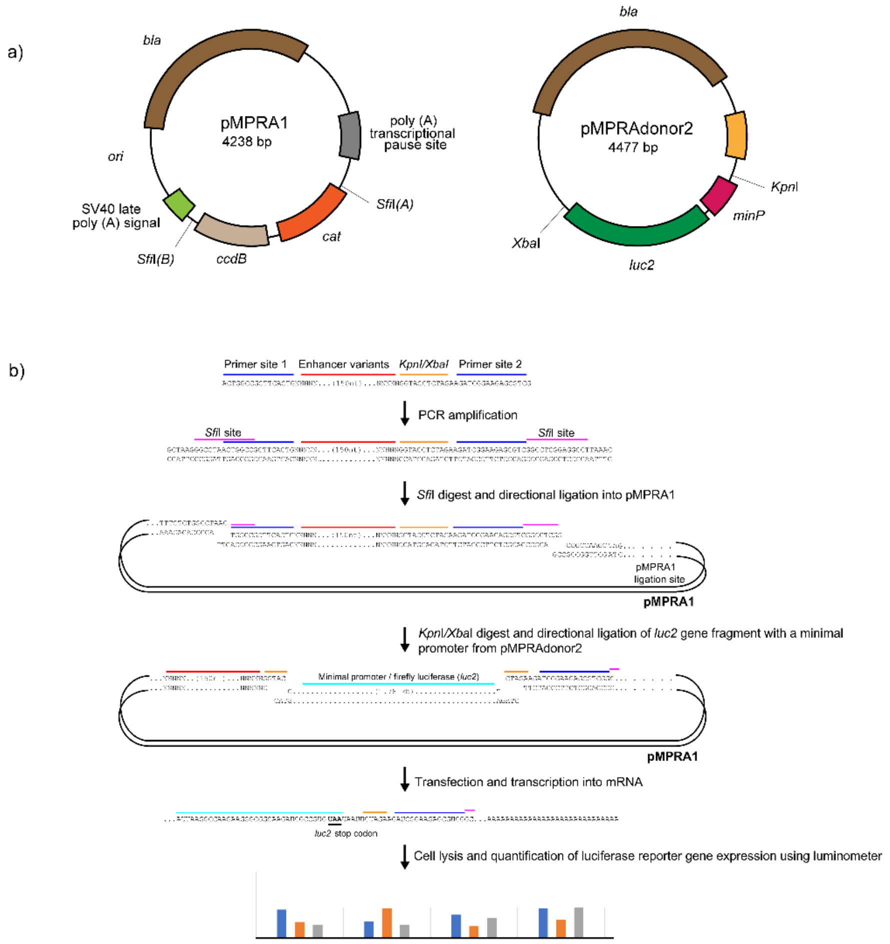


**Supplementary Figure 7. A flow chart of the plasmid-based reporter assay methodology.**

**(a)** Simplified maps of pMPRA1 and pMPRAdonor2 plasmid vectors. The pMPRA1 plasmid contains two *Sfi*I restriction sites for directional ligation of the oligonucleotide sequences. The pMPRAdonor2 contains *Kpn*I and *Xba*I restriction sites to facilitate directional ligation of the luciferase (*luc2*) reporter gene fragment, together with the minimal TATA-box promoter (minP) within the oligonucleotide sequences. **(b)** Oligonucleotide sequences used in the reporter assay are synthesised DNA sequences flanking the T1D SNPs (*i.e.* reference and alternative sequences) and containing a pair of *Kpn*I (GGTACC) and *Xba*I (TCTAGA) restriction sites for directional ligation of the reporter gene fragment. PCR amplification is performed to add two distinct *Sfi*I tails for directional ligation of the oligonucleotide sequences into the pMPRA1 backbone. Transformation of the plasmid containing the oligonucleotide sequences is performed in competent *E. coli* cells. The plasmid DNA is extracted from *E. coli* following successful transformation (*i.e.* presence of colonies). The extracted plasmid DNA is digested with *Kpn*I and *Xba*I, and the luciferase gene (*luc2*) fragment is ligated within the sequence. Transformation of the plasmid is again performed using competent *E. coli* cells. Following a successful transformation, plasmid DNA is extracted and sequenced to confirm the absence of indels. The plasmid DNA is transiently transfected into A549 and HepG2 cells, and luciferase activity is assessed after 48 hrs in a luminometer. This assay is a modification of [2]. Figure part **(a)** is modified from https://www.addgene.org/; and part **(b)** is redrawn from[3]. pMPRA1 – Addgene: plasmid #49349. pMPRAdonor2 – Addgene: plasmid #49353.

## Supplementary References


1. Karczewski, K. J. *et al.* The mutational constraint spectrum quantified from variation in 141,456 humans. *bioRxiv* 531210 (2019) doi:10.1101/531210.

2. Melnikov, A., Zhang, X., Rogov, P., Wang, L. & Mikkelsen, T. S. Massively parallel reporter assays in cultured mammalian cells. *J. Vis. Exp.* 1–8 (2014) doi:10.3791/51719.

3. Melnikov, A. *et al.* Systematic dissection and optimization of inducible enhancers in human cells using a massively parallel reporter assay. *Nat. Biotechnol.* **30**, 271–277 (2012).
