# Supplementary material for: Identifying the lungs as a susceptible site for allele-specific regulatory changes associated with type 1 diabetes risk
Source: Commun Biol. 2021 Sep 14;4:1072. doi: 10.1038/s42003-021-02594-0 (PMC8440780; doi:10.1038/s42003-021-02594-0)
Supplement: Supplementary file 3 — Description of Additional Supplementary Files [file 42003_2021_2594_MOESM3_ESM.pdf]

Description of Additional Supplementary Files

File name: Supplementary Data.

Description: Supplementary Data 1 – 14.

Supplementary Data 1: All genetic variants associated with the development of T1D used in the spatial eQTL analysis by CoDeS3D.

Supplementary Data 2: Hi-C libraries used in the analysis.

Supplementary Data 3: A summary of significant spatial eQTLs for T1D-associated SNPs analysed by CoDeS3D at FDR <0.05.

Supplementary Data 4: Significant enrichments for T1D eGenes (significance threshold <0.05.

Supplementary Data 5: Loss of function analysis for spatially regulated genes.

Supplementary Data 6: T1D-associated SNPs present within the Wellcome Trust Case Control Consortium (WTCCC) genotypes.

Supplementary Data 7: Tissue-specific weights from 50 model 1 predictors.

Supplementary Data 8: Lung contributions from the 50 model 1 predictors.

Supplementary Data 9: The lung eQTL (rs3087243-CTLA4) contribute an average of 4% to the disease risk.

Supplementary Data 10: AUC results of the validating model 2 on the 30 UK Biobank test dataset.

Supplementary Data 11: Ranking of eQTLs on tissue-specific contribution to T1D risk using T1D classification model 2.

Supplementary Data 12: Regulatory effects of rs6679677 from the blood eQTL database (http://www.eqtlgen.org)

Supplementary Data 13: Spatial eQTLs involving variants within the HLA locus.

Supplementary Data 14: Primer sequences used for DNA amplification and Sanger sequencing.
