# Supplementary material for: Identifying the lungs as a susceptible site for allele-specific regulatory changes associated with type 1 diabetes risk
Source: Commun Biol. 2021 Sep 14;4:1072. doi: 10.1038/s42003-021-02594-0 (PMC8440780; doi:10.1038/s42003-021-02594-0)
Supplement: Supplementary file 5 — Reporting Summary [file 42003_2021_2594_MOESM5_ESM.pdf]

nature portfolio

Corresponding author(s): Justin M. O'Sullivan COMMSBIO-20-2098A-Z

Last updated by author(s): 2021 08 02

Reporting Summary

Nature Portfolio wishes to improve the reproducibility of the work that we publish. This form provides structure for consistency and transparency in reporting. For further information on Nature Portfolio policies, see our Editorial Policies and the Editorial Policy Checklist.

Statistics

For all statistical analyses, confirm that the following items are present in the figure legend, table legend, main text, or Methods section.

n/a	Confirmed	
☐	☒	The exact sample size (*n*) for each experimental group/condition, given as a discrete number and unit of measurement
☐	☒	A statement on whether measurements were taken from distinct samples or whether the same sample was measured repeatedly
☐	☒	The statistical test(s) used AND whether they are one- or two-sided<br>*Only common tests should be described solely by name; describe more complex techniques in the Methods section.*
☒	☐	A description of all covariates tested
☒	☐	A description of any assumptions or corrections, such as tests of normality and adjustment for multiple comparisons
☒	☐	A full description of the statistical parameters including central tendency (e.g. means) or other basic estimates (e.g. regression coefficient) AND variation (e.g. standard deviation) or associated estimates of uncertainty (e.g. confidence intervals)
☒	☐	For null hypothesis testing, the test statistic (e.g. *F*, *t*, *r*) with confidence intervals, effect sizes, degrees of freedom and *P* value noted<br>*Give P values as exact values whenever suitable.*
☐	☒	For Bayesian analysis, information on the choice of priors and Markov chain Monte Carlo settings
☒	☐	For hierarchical and complex designs, identification of the appropriate level for tests and full reporting of outcomes
☒	☐	Estimates of effect sizes (e.g. Cohen's *d*, Pearson's *r*), indicating how they were calculated

Our web collection on statistics for biologists contains articles on many of the points above.

Software and code

Policy information about availability of computer code

Data collection	Python version 3.7.3 was used for all the python scripts. PLINK (v1.90b6.2, 64-bit) and bcftools version: 1.9 were used for SNP data cleaning and processing.
Data analysis	All statistical testing was performed using R software (version v3.6.3) 47, Scikit-learn (version 0.21.3; 46), tsfresh (version 0.12.0 44), and pymc3 (version 3.8; 48). Visualization for the luciferase luminescence was performed using GraphPad Prism (v8.4.3). Python version 3.7.3 was used for all the python scripts. Python scripts used used for machine learning are available at: https://github.com/Genome3d/ T1D_logistic_lasso_predictor.git/. CoDeS3D pipeline for tissue-specific eQTLs mapping is available at: https://github.com/Genome3d/codes3d-v1.

For manuscripts utilizing custom algorithms or software that are central to the research but not yet described in published literature, software must be made available to editors and reviewers. We strongly encourage code deposition in a community repository (e.g. GitHub). See the Nature Portfolio guidelines for submitting code & software for further information.

Data

Policy information about availability of data

All manuscripts must include a data availability statement. This statement should provide the following information, where applicable:

- Accession codes, unique identifiers, or web links for publicly available datasets
- A description of any restrictions on data availability
- For clinical datasets or third party data, please ensure that the statement adheres to our policy

All datasets generated for this study are included in the article as Supplementary Tables deposited with the source data of Figure 4 in figshare doi: 10.17608/k6.auckland.15071226. The genotype data from Wellcome Trust Case and Control Consortium and UK Biobank are restricted to share by the data sharing

agreements. Please refer to the organizations for obtaining the genotype data.

Field-specific reporting

Please select the one below that is the best fit for your research. If you are not sure, read the appropriate sections before making your selection.

- [x] Life sciences
- [ ] Behavioural & social sciences
- [ ] Ecological, evolutionary & environmental sciences

For a reference copy of the document with all sections, see nature.com/documents/nr-reporting-summary-flat.pdf

Life sciences study design

All studies must disclose on these points even when the disclosure is negative.

Sample size	Genotypes from Type 1 diabetes cases (2000) and controls (3000) were obtained from the Wellcome Trust Case Control Consortium.
Data exclusions	PLINK (v1.90b6.2, 64-bit) was used for quality control. Genotypes were cleaned using the Method-of-moments F coefficient estimate to remove homozygosity outliers (F values < -0.04 or 0.025 < F values). Related individuals were identified and removed using proportion IBD (PI_HAT > 0.08). Ancestry outliers (identified by principal component analysis [PCA] plotting), individuals with sex genotype errors (identified by PLINK), or individuals with missing genotype data (missing rate > 5%) were also removed. Finally, SNPs that were not in Hardy-Weinberg Equilibrium (p < 10-6) or had a minor allele frequency < 1% were removed before SNP data imputation (Sanger imputation server; https://imputation.sanger.ac.uk)43. Following imputation, the T1D genotype data was cleaned to remove SNPs with an: impute2 score < 0.3; missing data rate > 5%; or a minor allele frequency < 1%.
Replication	Data security and code management is the foundation of reproducible and reliable data analyses. Datasets that were received or downloaded from original sources were individually maintained in read-only and write-protected directories on secured cloud server. Program code was preserved after producing validated results and the code was named with appropriate functional and step sequential information. Version control (git) was also employed to protect script integrity across the analysis step directories.
Randomization	The WTCCC genotype derived T1D eQTL matrix was randomly split (80:20) into two groups that contained case and control genotype data for prediction model training and validation.
Blinding	Investigators were blinded to the data allocation performed by python numpy-permutation in the training and validation datasets.

Reporting for specific materials, systems and methods

We require information from authors about some types of materials, experimental systems and methods used in many studies. Here, indicate whether each material, system or method listed is relevant to your study. If you are not sure if a list item applies to your research, read the appropriate section before selecting a response.

Materials & experimental systems

n/a	Involved in the study	
[x]	[ ]	Antibodies
[ ]	[x]	Eukaryotic cell lines
[x]	[ ]	Palaeontology and archaeology
[x]	[ ]	Animals and other organisms
[x]	[ ]	Human research participants
[x]	[ ]	Clinical data
[x]	[ ]	Dual use research of concern

Methods

n/a	Involved in the study	
[x]	[ ]	ChIP-seq
[x]	[ ]	Flow cytometry
[x]	[ ]	MRI-based neuroimaging

Eukaryotic cell lines

Policy information about cell lines

Cell line source(s)	A549 (lung epithelial carcinoma; ATCC); HepG2 (human liver carcinoma; ATCC)
Authentication	The cell lines were purchased directly from ATCC (American Type Culture Collection) and used at an early passage number.
Mycoplasma contamination	All cell lines tested negative for mycoplasma contamination.
Commonly misidentified lines (See ICLAC register)	No commonly misidentified cell lines were used in the study.
